# Assessing the impact of the Lebanese National Polio Immunization Campaign using a population-based computational model

**DOI:** 10.1186/s12889-017-4909-0

**Published:** 2017-11-25

**Authors:** Ali Alawieh, Zahraa Sabra, E. Farris Langley, Abdul Rahman Bizri, Randa Hamadeh, Fadi A. Zaraket

**Affiliations:** 1 Department of Microbiology and Immunology, Medical University of South Carolina, Charleston, SC USA; 2 Department of Neurosciences, Medical University of South Carolina, Charleston, SC USA; 3 Electrical and Computer Engineering Department, Maroun Semaan Faculty of Engineering and Architecture, American University of Beirut, Beirut, Lebanon; 4 Department of Internal Medicine, Division of Infectious Diseases, American University of Beirut Medical Center, Beirut, Lebanon; 5 Head, Primary Health Care Department, Director of Immunization Program, Lebanese Ministry of Public Health, Beirut, Lebanon; 6 National Certification Committee, Polio Eradication Initiative - Lebanon, Beirut, Lebanon

**Keywords:** Poliomyelitis, Vaccine, Vaccination campaign, Syrian war, Statistical model

## Abstract

**Background:**

After the re-introduction of poliovirus to Syria in 2013, Lebanon was considered at high transmission risk due to its proximity to Syria and the high number of Syrian refugees. However, after a large-scale national immunization initiative, Lebanon was able to prevent a potential outbreak of polio among nationals and refugees. In this work, we used a computational individual-simulation model to assess the risk of poliovirus threat to Lebanon prior and after the immunization campaign and to quantitatively assess the healthcare impact of the campaign and the required standards that need to be maintained nationally to prevent a future outbreak.

**Methods:**

Acute poliomyelitis surveillance in Lebanon was along with the design and coverage rate of the recent national polio immunization campaign were reviewed from the records of the Lebanese Ministry of Public Health. Lebanese population demographics including Syrian and Palestinian refugees were reviewed to design individual-based models that predicts the consequences of polio spread to Lebanon and evaluate the outcome of immunization campaigns. The model takes into account geographic, demographic and health-related features.

**Results:**

Our simulations confirmed the high risk of polio outbreaks in Lebanon within 10 days of case introduction prior to the immunization campaign, and showed that the current immunization campaign significantly reduced the speed of the infection in the event poliomyelitis cases enter the country. A minimum of 90% national immunization coverage was found to be required to prevent exponential propagation of potential transmission.

**Conclusions:**

Both surveillance and immunization efforts should be maintained at high standards in Lebanon and other countries in the area to detect and limit any potential outbreak. The use of computational population simulation models can provide a quantitative approach to assess the impact of immunization campaigns and the burden of infectious diseases even in the context of population migration.

## Background

The Global Polio Eradication Initiative anticipated the eradication of wild-type poliovirus (WPV) in 2000. However, three foci of WPV, Afghanistan, Pakistan and Nigeria, still contribute to recurrent outbreaks of re-infection in areas of instability [1–4]. Two main forms of poliovirus, WPV1 and to a lesser extent WPV3, affect children in countries with low immunization coverage [5, 6]. The seriousness of this infection is due to potential fatality, occult spread, and high infectivity of the virus through fecal-to-oral route [4]. Recently, cases of WPV were reported from areas other than the three remaining foci due to WPV re-introduction in Somalia, northern Kenya [7], and Syria [8]. This incurs enormous costs on countries revisited by the virus mainly through immunization campaigns and management of affected cases [9].

The recent outbreak of poliomyelitis in Syria is a major concern due to difficulty in containment amid the conflict and the threat of importing the infection to neighboring polio-free countries like Lebanon [10, 11]. The Syrian outbreak is the first in 14 years and has resulted in initial detection of 17 cases in Deir ez-Zor and Aleppo [12]. The origin of the virus is likely Pakistan, and it is believed to be the same virus isolated from the sewage system in Israel, Egypt and Gaza [8, 13]. The severity and potential threat of this outbreak lead the WHO to launch the largest-ever consolidated immunization response in the Middle East to vaccinate around 22 million children repeatedly across seven different countries including Lebanon [10, 14, 15].

Lebanon has been polio-free since 1994 [16]; however, because of its proximity to Syria and the flow of large numbers of Syrian refugees, Lebanon was considered to be at risk of WPV reintroduction. Here, we reviewed the results of poliomyelitis surveillance in Lebanon and the outcome of the most recent national immunization campaigns. We propose a stochastic model of a potential polio outbreak and quantitatively evaluate the impact of the immunization campaign. Finally, we assess the risks and challenges of WPV re-emergence after the campaign.

## Methods

### Acute flaccid paralysis surveillance

Polio is a reportable disease in Lebanon and the implemented surveillance system monitors the infection through investigating all cases of acute flaccid paralysis (AFP) in children under 15 in accordance with WHO recommendations. The system includes three aspects of monitoring: obligatory reporting of AFP cases by physicians, weekly hospital zero-reporting, and active surveillance through hospital active sentinel systems. Records of all AFP patients were obtained from the Lebanese Ministry of Public Health (LMPH) that included comparable reporting from Lebanese, Syrian, and Palestinian populations in Lebanon [17].

### Status of resident population demographics in Lebanon

Three major subpopulations currently reside in Lebanon including Lebanese nationals, Syrian refugees, and Palestinian refugees. Numbers, demographics and geographical distribution of Syrian refugees were obtained from the United Nations High Commissioner for Refugees (UNHCR) [18]. Demographics of Lebanese nationals were acquired from the United Nations Development Program statistics (UNDP) [19].

### Stochastic model of potential polio outbreak

We developed a computational model customized to the Lebanese setting to assess the burden of potential polio outbreaks. We first generated a simulated population across the different Lebanese provinces. Then we simulated the introduction of Polio cases before and after the national polio vaccination campaign. The source-code and implementation of the model are available online at (https://github.com/alialawieh1/PolioModel).

#### Individual features definition

**Fig. 1:**
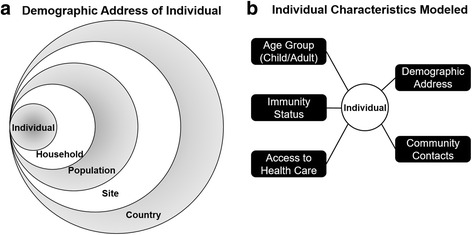
Features of simulated individuals. In (**a**), individuals are defined by a demographic address that include a specific household, a specific population (Lebanese, Palestinian or Syrian), a specific geographic site (Beirut, Mount Lebanon, Bekaa, North, South), and country (Lebanon). **b** shows the different features assigned to each individual in the population

Lebanon harbors two different refugee populations (Palestinian and Syrian) in addition to Lebanese nationals. Refugees are often segregated in camps with different epidemiological features compared to Lebanese nationals. Demographics of Lebanese residents are simulated based on data from the most recent UNDP statistics for Lebanese nationals and from UNHCR for refugees. The immunization status of the different populations and statistics on healthcare access were obtained through the LMPH [20]. In our model, we simulated Lebanese residents through a hierarchical structure (Fig. 1). Each individual is defined as vector of features *Ind* =  < *s*, *p*, *hc*, *h*, *n*, *i*, *a*, *m*> which denote the site or province, the subpopulation (Lebanese, Syrian, Palestinian), the household size, the household, the individual number, the immunization status (Fully, partially or not immune), the age group, and the medical access, respectively (Fig. 1).

#### Populations generation algorithm

Input data retrieved from public resources is summarized in Table 1. Demographic information provides the number of households ${NH}_{s,p}^j$with household size *j* for each subpulation *p* in each geographic site *s*. The subpopulation generation algorithm uses this information to construct a subpopulation hierarchial tree where the country is the root *v*_*r*_. First level nodes in the tree {*v*_1_, *v*_2_, …, *v*_*k*_} are all children nodes of *v*_*r*_ and define the *k* geographical sites in the country. Each v_s_ node corresponding to a geographical site *s* is parent to three subpopulation type nodes {*v*_*s*, *leb*_, *v*_*s*, *pal*_, *v*_*s*, *syr*_}. Each of the latter nodes is parent to household size nodes *v* ^*j*^_*sˎp*_ where *p* denotes a subpopulation and *jε*{1, 2, 3, 4, 5, 6, 7, 8} denotes the number of individuals in the household.

**Table 1:** Input Data on Population Distribution and Demographics

Variable		Estimate	Value	Reference
Total Population Estimate				
Lebanese Population*		Point	5,882,562	[19]
Syrian Refugee Population		Point	1,435,840	[18]
Palestinian Refugee Population		Point	455,000	[28]
Geographic Distribution				
Lebanese Population*	Beirut	Point	49.9%	[19]
	Bekaa	Point	12.9%	[19]
	North	Point	21.6%	[19]
	South	Point	15.7%	[19]
Syrian Refugee Population	Beirut	Point	29.5%	[18]
	Bekaa	Point	35.1%	[18]
	North	Point	24.7%	[18]
	South	Point	11.8%	[18]
Palestinian Refugee Population	Beirut	Point	16.4%	[28]
	Bekaa	Point	7.7%	[28]
	North	Point	21.0%	[28]
	South	Point	54.9%	[28]
Average Household Size Across Different Sites				
Beirut		Mean, Range	3.99, [1–8]	[19]
Bekaa		Mean, Range	4.58, [1–8]	[19]
North		Mean, Range	4.74, [1–8]	[19]
South		Mean, Range	4.38, [1–8]	[19]
Percentage of Adults (> 16 years) Among Populations				
Lebanese Population		Point	62.7%	[19]
Syrian Refugee Population		Point	46.8%	[18]
Palestinian Refugee Population		Point	67.2%	[28]
Polio Immunization Coverage Across Populations Prior to Immunization Campaign				
Lebanese Population	Adults (Fully Immune)	Point	91.0%	#
	Children (Fully Immune)	Point	95.0%	#
	Adults (Partially Immune)	Point	0.0%	#
	Children (Partially Immune)	Point	0.0%	#
Syrian Refugee Population	Adults (Fully Immune)	Point	95.0%	#, [2, 25]
	Children (Fully Immune)	Point	32.5%	#, [2, 25]
	Adults (Partially Immune)	Point	0.0%	#, [2, 25]
	Children (Partially Immune)	Point	47.5%	#, [2, 25]
Palestinian Refugee Population	Adults (Fully Immune)	Point	93.0%	#, [29]
	Children (Fully Immune)	Point	63.0%	#, [29]
	Adults (Partially Immune)	Point	0.0%	#, [29]
	Children (Partially Immune)	Point	12.5%	#, [29]
National immunization coverage post campaign		Point	97.8%	#
Access to Medical Care				
Lebanese Population		Point	98.0%	#,$
Syrian Refugee Population		Point	75.0%	[25]
Palestinian Refugee Population		Point	75.0%	#,$

*In absence of a governmental census in Lebanon, data from United Nations Development Programme was used as best estimate

# Data from recent reports of Lebanese Ministry of Public Health

$ Estimated using current data on acute flaccid paralysis surveillance and immunization level

Then, for each *v* ^*j*^_*sˎp*_, household-size node of subpopulation *p* and site *s,* the algorithm instantiates a total of ${NH}_{s,p}^j$children household nodes. Each ${v}_{s,p}^{h,j}$household node where $h\varepsilon \left\{1,2,\dots, {NH}_{s,p}^j\right\}$is in turn parent of *j* nodes representing individuals.

We designate by ${N}_{s,p}=\left\{{Ind}_1^{s,p},{Ind}_2^{s,p},{Ind}_{n_{s,p}}^{s,p}\right\}$the set of all individuals in subpopulation *p* and site *s*, and by *n*_*sˎp*_the cardinality of *N*_*sˎp*_.

This defines the *Ind* =  < *s*, *p*, *hc*, *h*, *n*, > features of all instantiated individuals. The algorithm then assigns values for the immunization status, age group and medical status features <*i*, *a*, *m*>of each individual ${Ind}_j^{s,p}$ where 1 < *j* < *n*_*s*, *p*_as follows.

The age group for each individual was assigned randomly while obeying the population’s age distribution. Based on the age group, the immunization and medical care statuses were assigned to respect the demographic distribution per site and uniform access to healthcare within each household. Because there is no apparent gender difference in susceptibility to poliovirus infection, we excluded data on gender distribution.

The output of this population construction algorithm is a set of vectors describing demographic, geographic, and immune features of each individual; *Ind* =  < *s*, *p*, *hc*, *h*, *n*, *i*, *a*, *m*, >.

#### Case features definition

A disease instant vector *Dis* includes five features <*Dur*, *LP*, *IP*, *SS*, *Inf*> denoting disease duration, latency period, incubation period, symptomatic status and infectivity, respectively.

Each infected individual during a simulated outbreak is defined as a concatenation vector (*PC*) of the individual vector (*Ind*)and a disease vector (*Dis*) that defines the properties of the infection instant.

Given that disease features are not point estimates, we used a skewed Gaussian distribution to determine each feature using population statistics by a disease instant characterization sequence (DICS). For each disease property such as latency and incubation, literature estimates are commonly reported as mean and range of values (Table 2). We developed a DICS sequence that retrieves relevant population statistics vector (*P*_*Dis*_(*Ind*) =  < *X*_*Dur*_, *X*_*LP*_, *X*_*IP*_, *X*_*SS*_, *X*_*Inf*_>) for each disease instant based on the patient’s properties defined in *Ind*. Each element *X*_*k*_ *εP*_*Dis*_(*Ind*), *kε*{*Dur*, *LP*, *IP*, *SS*, *Inf*} is a skewed Gaussian distribution defined by a mean $\overline{k}$, upper limit *k*_*H*_, and lower limit *k*_*l*_. Following the generation of the distributions, an instance from each distribution in *P*_*Dis*_(*Ind*) is assigned to the features of the *Dis* vector.

**Table 2:** Input Data on Disease and Individual Properties

Variable		Estimate	Value	Reference	Description
Latency Period		Mean, Range	2, [0.1–7]	[5, 26, 30, 31]	Duration before case becomes infectious
Incubation Period		Mean, Range	10, [0.1–20]	[5, 26, 30, 31]	Duration of infectivity prior to symptoms development
Disease Duration	Non-immune	Mean, Range	35, [20–50]	[5, 26, 30, 31]	Full duration of disease from latency to resolution or death
	Partially-immune	Mean, Range	7, [3–20]	[5, 26, 30, 31]	Full duration of disease from latency to resolution or death
Household infection rate	Partially-immune case	Mean, Range	0.1, [0 0.5]	[5, 26, 30, 31]	Probability that the individual spreads the infection among household members
	Non-immune	Mean, Range	1, [1 1]	[5, 26, 30, 31]	Probability that the individual spreads the infection among household members
Paralysis among cases, non-immune		Point	0.005	[5, 30, 31]	Probability of paralysis in non-immune cases
Paralysis among cases, partially-immune		Point	0	[5, 30, 31]	Probability of paralysis in partially-immune
Symptoms among cases, non-immune		Point	0.05	[5, 30, 31]	Probability of developing symptoms in the non-immune cases
Symptoms among cases, partially-immune		Point	0	[5, 30, 31]	Probability of developing symptoms in the partially-immune cases
Contacts per day	Child, within province	Mean, Range	2, [1–10]	*	
	Adult, within province	Mean, Range	2, [1–4]	*	
	Child, outside province	Mean, Range	1, [0–3]	*	
	Adult, outside province	Mean, Range	1, [0–3]	*	
Contact distribution across population	Same population	Point	66.70%	*	Percentage of individual contacts that belong to the same population as the case
	Different population	Point	33.30%	*	Percentage of individual contacts that do not belong to the same population as the case

*Estimated from the recommendations of Lebanese National Poliomyelitis Certification Committee (Dr. Bizri, Dr. Hamadeh)

The *inf* vector determines case infectivity where inf =  < inf_*H*_, inf_*P*_, inf_*O*_>is a vector of household infectivity, inf_*H*_ within-province infectivity inf_*P*_and outside-province infectivity inf_*O*_. This variable is used to determine the spread of infection and the propagation of the disease.

#### Simulating potential outbreak

To simulate an outbreak, single or multiple cases are defined by the user and introduced into one of the Lebanese provinces. The Monte Carlo based simulation draws a number of infected cases at time 0 and propagates the infection based on a hierarchical contact model that respects the following:

1. The latency and incubation period are simulated as Boolean constraints on infectivity,
2. The age group and immunization level are simulated as multiplier rates of infectivity, and
3. The household, within and outside province, and within and outside subpopulation contacts are simulated based on likelihood of establishing a close contact (Table 2).

Each case is assigned a disease vector. Contacts of the infected cases are drawn based on a hierarchical contact model (Fig. 1a). The non-immune or partially immune contacts of each infective case are evaluated for infectivity based on the infectivity distributions. The simulation proceeds on a day-by-day basis and keeps track of the number of infected, symptomatic, and detected cases.

The Monte Carlo simulation is also governed by the following rules:

1. A detected case cannot infect others as it will be isolated.
2. A symptomatic case with access to health care cannot infect others.
3. A paralyzed case cannot infect others.
4. All household members are closely connected.

Finally, the distributions are set such that children are more susceptible to initial infection, adults have more contacts outside the household, outside the province, and outside the subpopulation.

#### Evaluating the impact of national immunization campaign

The Lebanese national polio immunization campaign (NPIC) was launched in November 2013 after the Syrian polio outbreak. The approach, coverage and results of the two rounds of the immunization campaign were reviewed based on the data from the LMPH.

Results of the immunization campaign were used to re-run our model in order to evaluate the impact of the NPIC. To do this, we updated the values of the polio immunization levels among children, and the health care coverage of all individuals (Table 1), and performed similar simulations.

### Model implementation and data visualization

The model is implemented in MATLAB 2013a. By incorporating a random allocation step in population construction and case attributes assignment, the simulation can be run for several rounds to test consistency and allow for detecting variation due to random allocation. For that reason, each simulation is run for 1000 times and output is reported as average values with the standard deviation. The output shows the evolution and distribution of the cumulative number of infected individuals over time (in days) and the number of symptomatic, detected, paralyzed, and/or dead cases. A detected infection is defined as case who develops symptoms while having access to medical care.

### Statistical analyses

Inputs used in this model are based on national and international statistics for demographics and health statistics. Data is commonly reported as point estimate (e.g. number of households per site) or a range and mean of values (viral incubation period). Point estimates were used in the first case, and Gaussian distributions following the range and mean constrains were used in the second case. To avoid bias, the population generation algorithm was simulated for 1000 times for each scenario. Using 10,000 iterations did not add significant difference to the mean results (*p* > 0.05, t-test), and therefore 1000 iterations were used in all simulations. All statistical analyses were performed on Graphpad Prism 6.

### Limitations

The method used in this paper presents a novel approach to simulate outbreak propagation, and a new methodology to assess the efficacy of interventions such as vaccination campaigns. However, the presented model uses geographic and demographic inputs that are specific to Lebanon. Therefore, while the approach and the model can be executed with inputs relevant to countries other than Lebanon, the conclusions presented in the paper regarding vaccination threshold and outbreak progressions may not be applicable to other countries. Another limitation of this study is the lack of thorough census of Lebanese, Syrian and Palestinian populations in Lebanon, and the inputs used to the models are the best estimates from governmental and international agencies. Another limitation of the model is that infiltrating refugees, i.e. those not concentrated in camps, are under-represented in this model and are hard to account for in statistical studies. Finally, the model does not independently study the different strains of poliovirus, and does not account for emergence of circulating vaccine-derived poliovirus (cVDPV) which has recently appeared in Syria. Notably, the emergence of cVDPV in Eastern Syria occurred after the influx of refugees to Lebanon, and is not currently a major concern to Lebanon given that refugees are currently starting to leave Lebanon back to Syria.

## Results

### Records of AFP surveillance in Lebanon

Before 2013, an average of 16 AFP cases are investigated per year in Lebanon (Table 3 **)**, ranging from rom 0.79 to 2.34 per 100,000. Around 31.8% of these cases involved children less than 5 years old; the most susceptible group to poliomyelitis. However, between 2013 and 2015, 34 to 113 annual AFP cases were investigated with a rate ranging between 4.2 to 13.9 per 100,000. Of these reported AFP cases, 39 cases involved children less than 5 years old (Table 3). Only one case of AFP was confirmed as poliomyelitis in 2003, and further follow-up revealed that the case was imported.

**Table 3:** Results of AFP Surveillance in Lebanon 1998–2015

Year	AFP total case	Cases (Syrian refugees)	Cases in children (<5 years)	Investigation rate	Poliomyelitis Confirmed	Poliomyelitis Compatible
1998	10	0	5	90%	0	0
1999	13	0	5	100%	0	0
2000	13	0	5	100%	0	0
2001	14	0	5	100%	0	0
2002	16	0	3	100%	0	0
2003	20	0	4	100%	1^a^	0
2004	14	0	5	100%	0	0
2005	13	0	3	100%	0	0
2006	15	0	4	93%	0	0
2007	23	0	5	100%	0	0
2008	18	0	7	100%	0	1
2009	8	0	3	100%	0	0
2010	19	0	6	100%	0	0
2011	22	0	5	100%	0	0
2012	24	0	4	100%	0	0
Average	16.1	0	4.6	99%		
2013	34	7	20	100%	0	0
2014	50	23	19	100%	0	0
2015	113	10	32	100%	0	0

^a^Further Investigation revealed that this case was imported

### National polio immunization campaign in Lebanon

Four cycles of national immunization were carried out by the with the help of non-governmental and international organizations including WHO and UNICEF [21]. The campaign involved vaccinating of all Lebanese residents as well as incoming Syrian children through special centers at the Syrian-Lebanese borders starting in November 2013. The campaign covered children up to five years of age [21], and involved visiting refugee camps, schools, nurseries, and homes. This allowed for immunization of registered and non-registered refugees as well. The NPIC utilized the OPV vaccine that is provided by the WHO for free [21], which was replaced by the bivalent OPV (bOPV) in late 2014. By the end of 2015, the NPIC completed four vaccination cycles achieving coverage of 97% among Lebanese residents.

### A customized model of potential outbreaks

Using a computationally simulated model of the Lebanese population, we have developed a novel model to predict the burden of potential polio outbreaks in response to hypothetical cases imported across the Syrian border. We have considered a population of 5,882,562 Lebanese, 1,435,480 Syrian, 455,000 Palestinians based demographic inputs (Table 1). Individuals were distributed across four major sites: Beirut, North Lebanon, Bekaa and South Lebanon. Within each site, individuals were distributed among households of variable sizes depending on site-specific demographics. Hypothetical cases were then introduced to one or multiple sites to assess the burden of infection.

### Burden of potential polio outbreaks

**Fig. 2:**
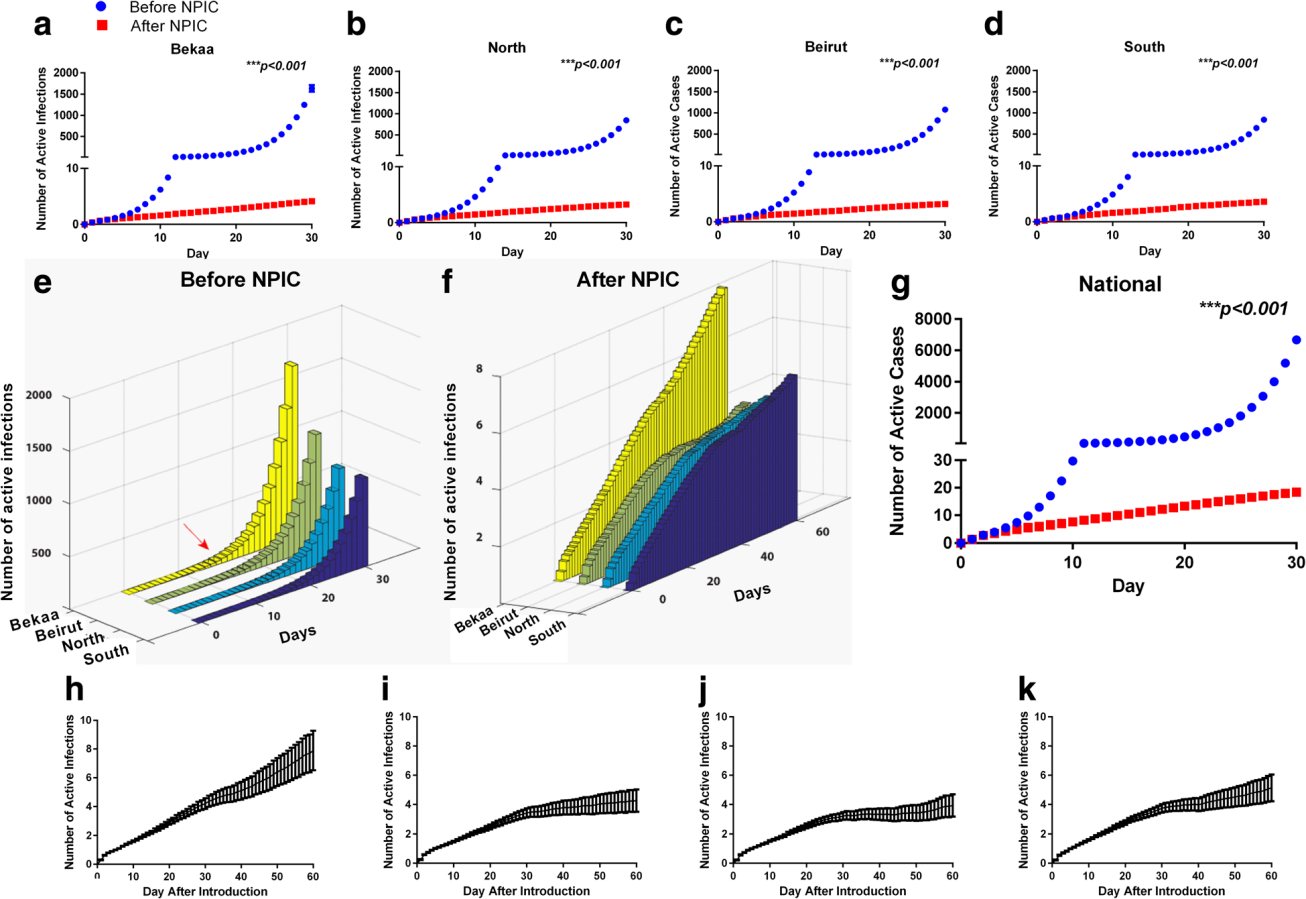
Simulation results of outbreak scenarios prior to and after the NPIC depending on site of first case introduction. **a**-**d** shows the cumulative number of infected cases in Lebanon within the first month after a single case is introduced to Bekaa, Beirut, North and South sites respectively. The NPIC significantly reduced the total number of infected individuals starting day 4 after the introduction (multiple t-test with 1% FDR, *p* < 0.001). **e**, **f** compares the different scenarios in (A-D) showing that an exponential increase in the number of cases is seen around 10 days after first case introduction regardless of the site only before the NPIC. **g** compares the evolution of number of cases before and after the NPIC assuming 5 cases of polio were simultaneously introduced to the country, 3 to Bekaa and 2 to North sites (multiple t-test with 1% FDR, *p* < 0.001). **h**-**k** shows the cumulative number of infected cases in Lebanon within the two months after a single case is introduced to Bekaa, Beirut, North and South sites respectively

We first simulated the introduction of a single Syrian case to different Lebanese provinces. Results show that prior to the NPIC, and regardless of the site of case introduction of the case, more than 1000 poliomyelitis infected individuals were expected to occur in Lebanon within 30 days of introduction (Fig. 2a-d). Comparing the different sites of introduction, a case introduced to Bekaa area, site of highest density of Syrian refugees, will result in a largest outbreak approaching near 2000 infected individuals by 30 days. We also noticed that around 12–13 days after initial introduction, the first order differential of accumulation curves approaches 2, the point at which cases start to at least double on a daily basis indicating an upcoming outbreak (Fig. 2e).

**Fig. 3:**
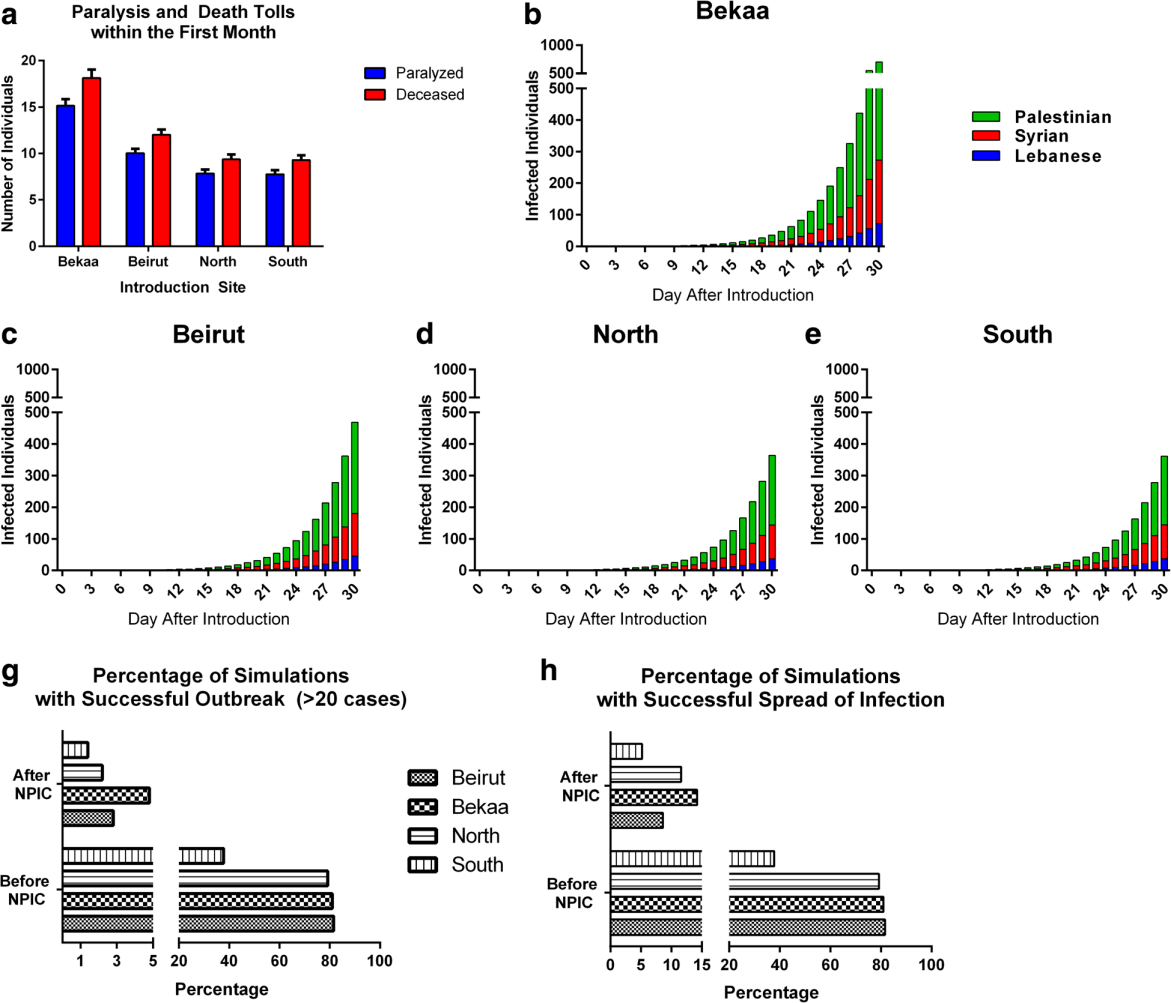
Burden and demographics of the outbreak prevented by the NPIC. **a** shows the anticipated number of patients who died or were paralyzed due to poliovirus infection in either of the four scenarios. **b**-**e** shows the distribution of infected cases across the three different resident populations in Lebanon given the four studied scenarios. **f** shows the percentage of simulations (among 1000 total simulations) in which polioviral infection propagates to at least one case after the initial case introduction before and after the NPIC. **g** shows the percentage of simulations (among 1000 total simulations) in which the introduced case results in an outbreak (defined as more than 20 cases within the first 30 days) before and after the NPIC

WPV infections may be asymptomatic or lead to manageable symptoms, paralysis or death. Therefore, we assessed the projected paralysis and/or death tolls resulting from the simulated outbreaks. During the first 30 days after case introduction, it is anticipated that, on average, 15 individuals will die and 10 will be paralyzed (Fig. 3a).

Interestingly, we assessed the distribution of cases across the different resident populations in Lebanon showing that although the hypothetical case is introduced in a Syrian community, the largest number of infected individuals is among the Palestinian refugee population regardless of site of introduction (Fig. 3b-e). In addition, we noticed minimal involvement of Lebanese residents in potential outbreaks compared to other populations (less than 10% of all cases).

### Assessment of impact of the NPIC

To assess the impact of the NPIC, we re-simulation the population while updating the polio vaccination coverage using recent data from the LMPH. At any site of case introduction, the cumulative number of infected individuals was significantly lower than that anticipated before the NPIC (*p* < 0.001, Fig. 1a-d), and did not exceed 10 infected individuals in the country over an extended period of 60 days after initial case introduction (Fig. 2f, h-k). With the exception of Bekaa site, propagation of number of infected individuals reached a plateau at around five infected individuals within 30 days. Introducing a case into Bekaa did not result in a similar plateau, but follow-up simulations for 120 days showed less than 10 infected individuals (not shown). We then investigated if the effects of the NPIC may be bypassed by the introduction of a larger number of cases to the country through simulating the entry of five different cases (two to North and three to Bekaa sites). Results show that the NPIC is still effective in significantly reducing the impact of potential outbreaks (Fig. 2g). The NPIC significantly reduced the projected number of infected individuals from around 8000 to less than 20 within 30 days (p < 0.001).

We assessed the probability that an outbreak will occur in 1000 simulations and we noticed that the NPIC have reduced the probability of development of outbreak or the spread of infection by around 15 folds (Fig. 3f, g). In addition, through our 1000 simulations, there were no cases of death or paralysis anticipated after the NPIC.

### Safety threshold of immunization coverage

**Fig. 4:**
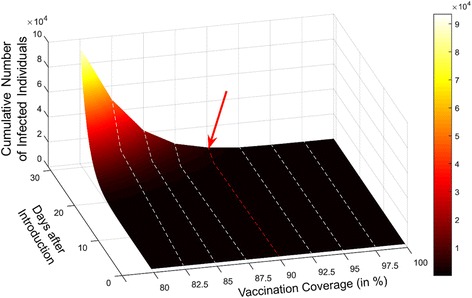
A 3-D plot of the cumulative number of infected individuals per time at different national immunization level. The curve shows that there is a high risk of exponentially growing outbreaks of poliomyelitis when the percentage of vaccinated individuals in Lebanon falls below 90%. Above this level, the evolution of a potential outbreak will become slow decreasing the probability that the virus will circulate for a long time and adopt new hosts

To determine the minimum vaccination coverage required to maintain a low risk of poliomyelitis outbreak in Lebanon, we simulated evolution of potential outbreaks at different national vaccination levels. Figure 4 shows the cumulative number of infected individuals with time at a range of national vaccination coverage between 80% and 100%, and indicates that below a threshold of 90% coverage, there is a risk of outbreak progression with more severe outbreak expected as the vaccination level drops to 80%.

## Discussion

Following its certification as a polio-free country, the Lebanese government has implemented a routine immunization protocol using a primary dose of Inactivated Polio Vaccine followed by two doses of Oral Polio Vaccine, the latter being also used for subsequent doses [17]. The LMPH has adopted a syndromic approach for AFP surveillance with complete investigation of reported cases. Results of AFP surveillance show the absence of any WPV infection over the past 15 years (Table 3). Starting 2013, the Lebanese healthcare system has witnessed several challenges associated with the presence of large numbers of Syrian refugees in the country [22, 23] including the threat of polio re-emergence after the Syrian outbreak [4, 15]. This led to a higher number of AFP cases reported in 2013 and 2014 (34 and 50 cases) due to the higher index of suspicion among medical practitioners.

The threat of WPV has driven the Lebanese government to implement measures to prevent a potential outbreak by boosting the underlying medical infrastructure including surveillance, prevention and case management [21]. Starting November 2013, the LMPH beefed up active surveillance in refugee areas and launched a four-phase national polio immunization vaccine (NPIC). Results from the campaign show coverage of around 98.4% of the target population. The significance of this campaign lies in the role of active immunization in halting the transmission cascade of WPV and preventing or reducing the burden of any potential outbreak [4, 24]. The importance of vaccination is evident in the Syrian scenario where first ten confirmed cases were found not to have been fully vaccinated against WPV [14].

We implemented a computational model of the Lebanese population to predict the consequences of a potential poliovirus outbreak. We show that the propagation of potential outbreaks before completing the NPIC was alarming (Fig. 2), and reflects the actual status of fear among healthcare professionals in the country and the Middle East. A major reason behind the high predicted burden of an outbreak is the low immunization coverage among Syrian children especially that field surveys have reported than 33% coverage in Syrian children what allows the virus to circulate for a longer period [24]. However, after the completion of the NPIC, the burden of a putative outbreak is extremely diminished. Based on our model, we have shown that regardless of the site of WPV introduction, the NPIC had a significant impact in reducing the risk of outbreak occurrence as well as nearly eliminating the risk of death or paralysis. Interestingly, our model emphasized the risk of disease progression in the Palestinian refugee population, the population with largest anticipated number of infected individuals. This prediction is relevant to the low healthcare coverage and worsening socioeconomic conditions across Palestinian refugee camps, a major target of incoming Syrian refugees. Another interesting finding is that after the NPIC, it is anticipated that the number of infected cases reaches a plateau around an average of 5 cases in the different introduction sites except for the Bekaa site. This finding matches the current high-risk profile of this area that houses the majority of Syrian refugees and is at the same time at a low healthcare and socioeconomic status.

Based on the anticipated propagation of infection in the country, our study emphasizes that with the continuous risk of incoming poliomyelitis cases from Syria, a minimum of 90% vaccination coverage needs to be maintained across the Lebanese resident populations to prevent the risk of future outbreaks. This level is currently achieve by the NPIC explaining, thus validating our model and explaining why no cases of WPV have yet been reported in Lebanon. However, a continuous effort from the Lebanese government, the health care community and international organization is still required to maintain awareness and immunization coverage in the entire community especially in underserved locations. Notably, this is faced by many challenges that include the ever deteriorating status of the healthcare system in Syria, the massive population movement across borders including illegal routes that are not monitored by border surveillance, the low levels of immunization in certain areas with incomplete governmental coverage, the high numbers of unregistered refugees, and most importantly compromised hygiene and sanitary conditions in refugee tented settlements or makeshift camps [2, 4, 10, 12, 25].

Several previous models of poliomyelitis spread and dynamics were previously employed to assess the dynamics of spread of the virus in a population setting and used mathematical approximation techniques and individual based models [26, 27]. Compared to these models, the model outlined in this manuscript uses similar inputs but provides a real-life simulation of the studied population characteristics and assesses interactions and transmissions at the individual level in contrast to approaches that approximate individual behavior at the population level [26, 27]. Our approach allows also for repetitive simulations to reach a more accurate prediction of what actual epidemiological scenarios may look like. Notably, although the findings of this model cannot be readily translated to nearby countries in the region given the unique design of the model to fit the Lebanese resident populations, the approach implemented in this model can be optimized and translated to predict efficacy of immunization campaigns and disease progression in other countries.

## Conclusions

This work describes a novel approach to predict poliovirus progression in Lebanon using simultaneous modeling of the population demographics and the disease propagation. We have also shown that the use of computational population simulation models can be expanded beyond the prediction of disease burden to provide a quantitative approach to assess the impact of immunization campaigns even in the context of population migration. Although the threat of virus introduction to Lebanon has significantly decreased given the minimal influx of refugees to Lebanon since 2016, the Lebanese government should continue to enhance surveillance of suspected poliovirus infections, and adopt supplementary surveillance activities such as collecting samples from sewage systems and from contacts of AFP cases. Furthermore, this last outbreak, along with previous similar ones in areas of conflict, re-emphasizes the importance of total eradication of polio from the remaining pockets. One major pre-requisite for that is restoring peace and tranquility.

## Abbreviations

AFP: Acute Flaccid Paralysis
DICS: Disease Instant Characterization Sequence
LMPH: Lebanese Ministry of Public Health
NPIC: National Poliovirus Immunization Campaign
WPV: Wild-type polioviruss
